# Concentration of acrylamide in a polyacrylamide gel affects VP4 gene coding assignment of group A equine rotavirus strains with P[12] specificity

**DOI:** 10.1186/1743-422X-7-136

**Published:** 2010-06-23

**Authors:** LaShanda M Long-Croal, Xiaobo Wen, Eileen N Ostlund, Yasutaka Hoshino

**Affiliations:** 1 Rotavirus Vaccine Development Section, Laboratory of Infectious Diseases, NIAID, National Institutes of Health, Bethesda, MD 20892, USA; 2 Center for Devices and Radiological Health, Food and Drug Administration, Silver Spring, MD 20994, USA; 3 Diagnostic Virology Laboratory, National Veterinary Services Laboratories, Animal and Plant Health Inspection Service, USDA, Ames, IA 50010, USA

## Abstract

**Background:**

It is universally acknowledged that genome segment 4 of group A rotavirus, the major etiologic agent of severe diarrhea in infants and neonatal farm animals, encodes outer capsid neutralization and protective antigen VP4.

**Results:**

To determine which genome segment of three group A equine rotavirus strains (H-2, FI-14 and FI-23) with P[12] specificity encodes the VP4, we analyzed dsRNAs of strains H-2, FI-14 and FI-23 as well as their reassortants by polyacrylamide gel electrophoresis (PAGE) at varying concentrations of acrylamide. The relative position of the VP4 gene of the three equine P[12] strains varied (either genome segment 3 or 4) depending upon the concentration of acrylamide. The VP4 gene bearing P[3], P[4], P[6], P[7], P[8] or P[18] specificity did not exhibit this phenomenon when the PAGE running conditions were varied.

**Conclusions:**

The concentration of acrylamide in a PAGE gel affected VP4 gene coding assignment of equine rotavirus strains bearing P[12] specificity.

## Background

Diarrheal disease is one of the principal causes of morbidity and mortality among young children in the developing world. Infectious diarrhea of neonatal animals is also one of the most common and economically devastating conditions encountered in the animal agriculture industry. Among an array of infectious agents including bacteria, viruses and parasites, group A rotaviruses are the single most important etiologic agents of diarrhea in infants and young children worldwide and in addition, they are the most commonly identified viral cause of diarrhea in neonatal food animals [1-4]. In 1975, rotaviruses were first demonstrated being involved in foal diarrhea [5], and later established as the major cause of diarrhea in young foals [6-8].

The genome of group A rotavirus, a member of *Reoviridae *family, consists of eleven segments of double-stranded RNA numbered 1-11 according to their order of migration in polyacrylamide gels, segment 1 being the slowest and segment 11 the fastest [9]. The rotavirus genome encodes six structural (VP1-VP4, VP6 and VP7) and six nonstructural (NSP1-NSP6) proteins [3]. Since two outer capsid proteins VP7 and VP4 are independent neutralization and protective antigens, a binary system of classification and nomenclature to designate the two neutralization specificities has been adopted: VP7 or G (because VP7 is a glycoprotein) serotype and VP4 or P (because VP4 is protease-sensitive) serotype [3]. Since (i) antibodies to the VP7 and VP4 have been demonstrated to confer resistance to virulent rotavirus in a type-specific manner in experimental animals; and (ii) observations made in various rotavirus vaccine trials have suggested that the induction of serotype-specific immunity may be important for optimal protection, serotypic-genotypic analyses of the VP7 and VP4 of a rotavirus derived from various animal species have been performed [3,10,11]. Such studies have established at least 14 G serotypes (21 G genotypes) and 14 P serotypes (29 P genotypes) [12]. Among equine rotaviruses, five G types (G3, G5, G10, G13 and G14) and three P types (P[7], P[12] and P[18]) have been identified.

In general, each rotavirus strain displays a dsRNA migration pattern (electropherotype) on polyacrylamide gels distinct from that of other strains [9,13]. Hence analysis of such genomic polymorphism as determined by polyacrylamide gel electrophoresis (PAGE) as well as gene sequencing have been routinely used for gene coding assignments. Such studies have established that the VP7 protein is encoded by genome segment 7, 8 or 9 depending upon the rotavirus strain. For example, the VP7 is encoded by the 7^th ^segment of rhesus rotavirus MMU18006 strain in a 12% gel [14], the 8^th ^segment of human rotavirus DS-1 strain in a 7.5% gel [15], and the 9^th ^segment of human rotavirus Wa strain in a 12% gel [16]. With regard to the VP4 protein, on the other hand, it is universally acknowledged that it is encoded by the genome segment 4 regardless of the rotavirus strain. During the course of generating various single gene substitution reassortants and hyperimmune antisera to them in an attempt to characterize and establish VP4 serotypes of selected equine rotaviruses [17], we found unexpectedly that the VP4 gene of equine rotavirus strains H-2, FI-14 and FI-23 was not the fourth segment but the third segment as determined by a standard 12% PAGE.

## Results and discussion

### Concentration of acrylamide affects the relative position of VP4 gene of equine rotavirus strains H-2, FI-14 and FI-23 in a PAGE gel

**Figure 1:**
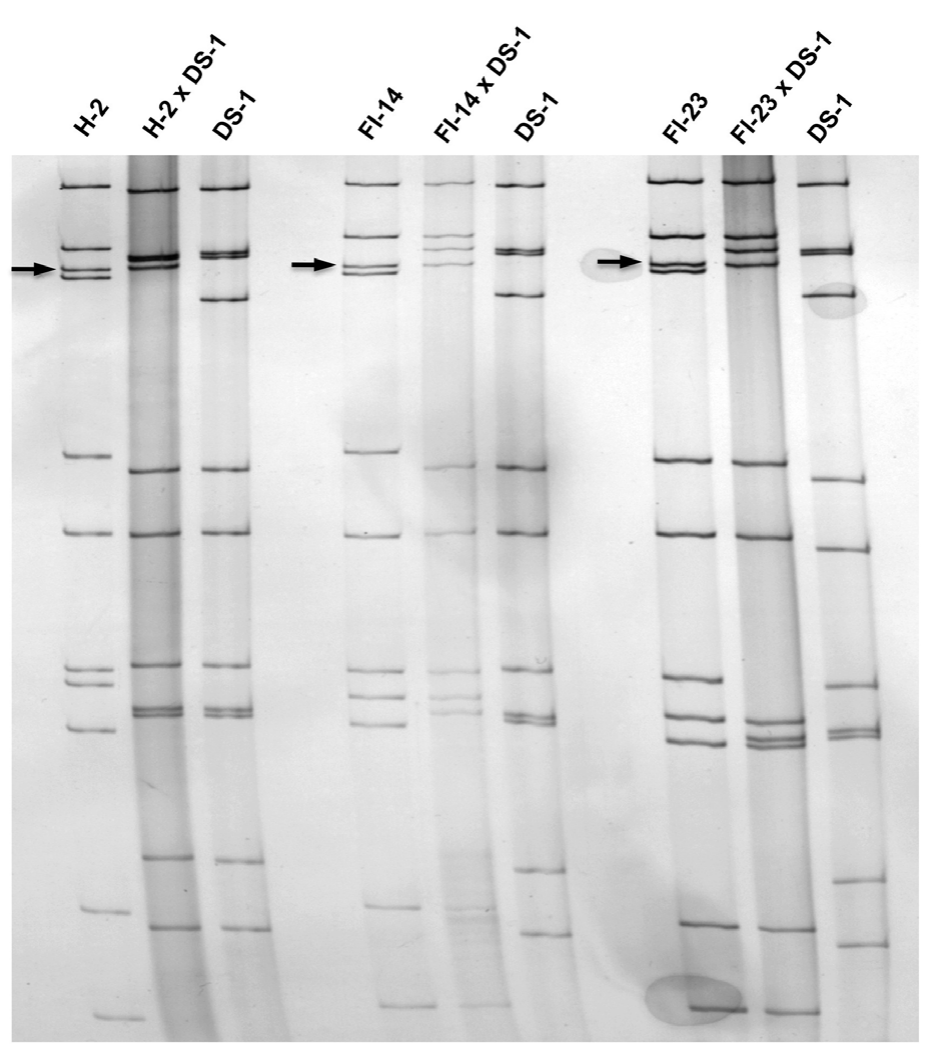
**Electrophoretic migration patterns in a 12% PAGE gel of equine rotavirus H-2 strain, H-2 × DS-1 reassortant, and human rotavirus DS-1 strain; equine rotavirus FI-14 strain, FI-14 × DS-1 reassortant and DS-1 strain; and equine rotavirus FI-23 strain, FI-23 × DS-1 reassortant, and DS-1 strain**. Arrows indicate the VP4 gene (3^rd ^genome segment) of each of the 3 equine parental rotavirus strains.

**Figure 2:**
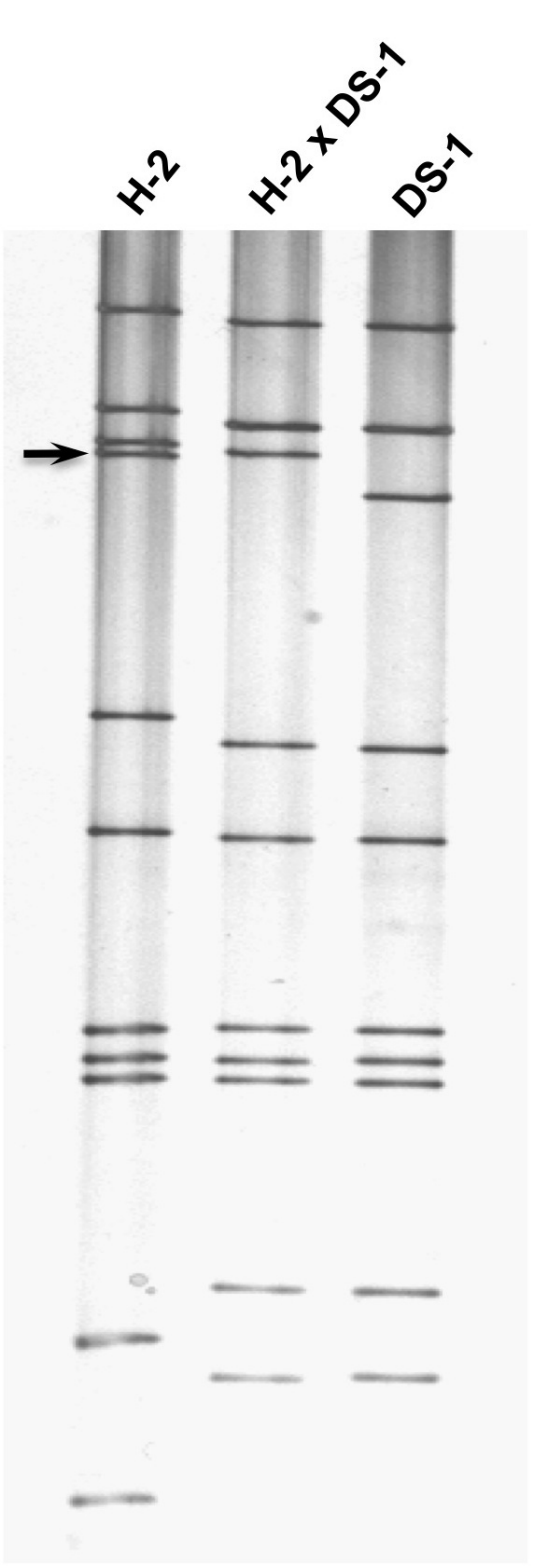
**Electrophoretic migration patterns in a 7.5% PAGE gel of equine rotavirus H-2 strain, H-2 × DS-1 reassortant and human rotavirus DS-1 strain**. Arrow indicates the VP4 gene (4^th ^genome segment) of the H-2 strain.

**Figure 3:**
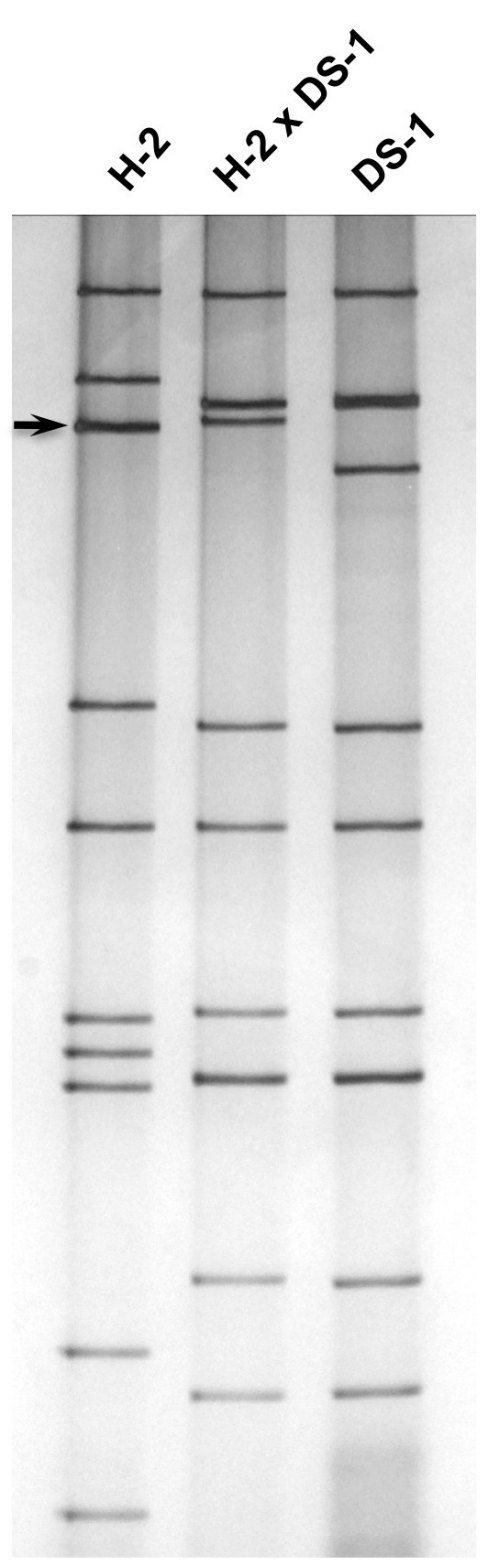
**Electrophoretic migration patterns in a 10% PAGE gel of equine rotavirus H-2 strain, H-2 × DS-1 reassortant and human rotavirus DS-1 strain**. Arrow indicates the VP4 gene of the H-2 strain. Note the 3^rd ^and 4^th ^genome segments of the H-2 strain comigrate.

**Figure 4:**
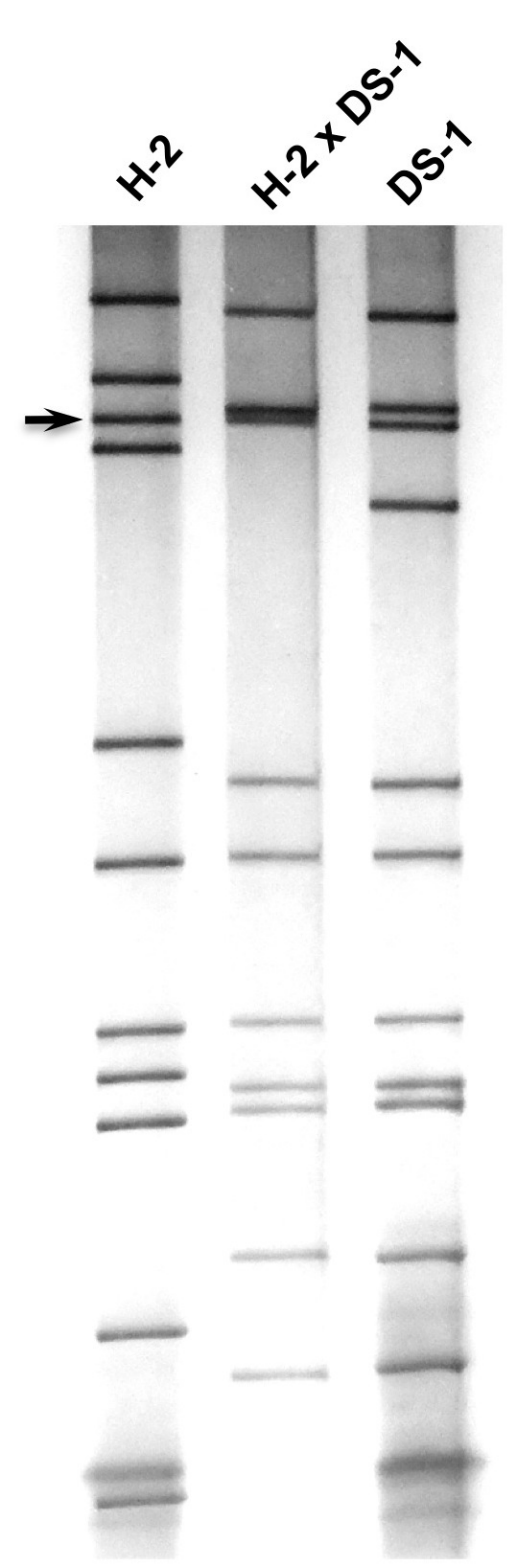
**Electrophoretic migration patterns in a 15% PAGE gel of equine rotavirus H-2 strain, H-2 × DS-1 reassortant and human rotavirus DS-1 strain**. Arrow indicates the VP4 gene (3^rd ^genome segment) of the H-2 strain.

During the characterization by a standard 12% PAGE gel analysis of selected equine-human rotavirus reassortants that were generated between equine rotavirus (strain H-2 [18], FI-14 [19] or FI-23 [20]) and human rotavirus (strain DS-1 [21]), we noticed that the VP4-encoding gene of each of the three equine rotavirus strains was at the third position (Figure 1). This was unexpected since the fourth genome segment was the VP4-encoding gene of human rotavirus strains Wa (P[8]) [21], DS-1 (P[4]), ST3 (P[6]) [22] as well as rhesus rotavirus strain MMU18006 (P[3]) [23] under the same PAGE running condition. Since we reported previously that the acrylamide concentration in a PAGE gel affected the relative position of the VP7 gene of G2 rotavirus strains [24], we analyzed the effects of acrylamide concentration by using H-2 strain and its reassortant rotavirus strain. The VP4 gene of the H-2 strain was in the 4^th ^position in a 5% (not shown) or 7.5% (Figure 2) gel, the 3^rd ^or 4^th ^poison in a 10% (Figure 3) gel, however, it was in the 3^rd ^position in a 12% (Figure 1) or 15% (Figure 4) gel. These findings demonstrated that the H-2 VP4 gene "flipped over" (i.e., the H-2 VP4 gene shifted to the 3^rd ^position from its previous 4^th ^position) in a PAGE gel containing acrylamide concentration between 7.5% and 12% (Table 1). Similarly, the FI-14 and FI-23 VP4 genes exhibited the "flip over" phenomenon between a 7.5% gel and a 12% gel (not shown, summarized in Table 1). Thus, we demonstrated that the concentration of acrylamide played a critical role in determining the VP4 gene coding assignment of equine rotavirus strains H-2, FI-14 and FI-23. As we reported previously, the different PAGE running conditions affected not only the VP4 gene but also other genes as well. For example, although segments 2 and 3 of the DS-1 strain comigrated in a 7.5% gel (Figure 2), they were well separated in a 15% gel (Figure 4).

**Table 1:** The concentration of acrylamide affects VP4-gene coding assignment of group A equine rotavirus strains H-2, FI-14, and FI-23 bearing P[12] specificity.

Rotavirus			Species of origin	VP4-gene coding assignment in a PAGE gel containing acrylamide at indicated concentration				
		
Strain [ref.]	P (VP4)type	G (VP7)type		5%	7.5%	10%	12%	15%
H-2 [18]	P[12]	G3	horse	4	4	3 or 4	3	3
FI-14 [19]	P[12]	G3	horse	ND^a^	4	ND	3	ND
FI-23 [20]	P[12]	G14	horse	ND	4	ND	3	ND
H-1 [25]	P[7]	G5	horse	4	4	4	4	4
L338 [26]	P[18]	G13	horse	4	4	4	4	4
Wa [21]	P[8]	G1	human	4	4	4	4	4
DS-1 [21]	P[4]	G2	human	4	4	4	4	4
ST3 [22]	P[6]	G4	human	4	4	4	4	4
MMU18006 [23]	P[3]	G3	rhesus	4	4	4	4	4

^a^ND = not done

### VP4 gene encoding P[12] specificity appeared to be affected most by the concentration of acrylamide in a PAGE gel

Next, we investigated whether the "flip-over" phenomenon was unique to P[12] equine rotavirus strains or common to any equine rotavirus strains. Previously [24], we showed that the VP4 gene of human rotavirus strains Wa (P[8]), DS-1 (P[4]), ST3 (P[6]) or rhesus rotavirus strain MMU18006 (P[3]) was at the 4^th ^position regardless of acrylamide concentration in a PAGE gel (Table 1). We found in this study that the relative position of the VP4 gene of equine rotavirus strain H-1 [25] with P[7] specificity and strain L338 [26] with P[18] specificity was not affected by the varying concentration of acrylamide in a PAGE gel (data not shown, summarized in Table 1). Thus, the "flip-over" phenomenon of the VP4 gene observed in the present study appeared to be unique to equine rotavirus VP4 genes bearing P[12] specificity.

The mechanisms underlying this "flip-over" phenomenon displayed by the VP4 gene with P[12] specificity are unknown. Since the observed VP4 gene migration shift appears to be a function of acrylamide concentration (all other factors being equal), this would indicate the size of the pores in the gel is what is generating the shift. This argues for the shift being the result of a change in the tertiary structure of the molecule. Unfortunately, tools do not exist at present for predicting secondary or tertiary structures for double-stranded nucleic acid sequences. We analyzed predicted secondary structures of single-stranded RNA of VP4 gene of selected rotavirus strains including equine rotavirus strains with P[12] specificity, however, we did not find any predicted structures that were different between the equine VP4 sequences and the others (data not shown). In addition, we examined the VP4 sequences of selected rotavirus strains to look for potential changes in the equine VP4 sequence that might induce some sort of "pairing" of the ends of the molecule, however, we did not find any good candidate sequences.

## Conclusions

The relative position of the VP4 gene of three equine P[12] strains (H-2, FI-14, FI-23) varied (either genome segment 3 or 4) depending upon the concentration of acrylamide. The VP4 gene bearing P[3], P[4], P[6], P[7], P[8] or P[18] did not exhibit this phenomenon when the PAGE running conditions were varied. Caution needs to be exercised when PAGE analyses are used for VP4 gene coding assignment of rotaviruses.

## Methods

### Rotavirus strains, cell culture, and genetic reassortment

Table 1 summarizes group A human and animal rotavirus strains that were employed in this study. Each of the rotavirus strains used was plaque purified three times prior to use. Reassortant rotaviruses between equine rotavirus strain H-2, FI-14 or FI-23 and human rotavirus strain DS-1 were constructed by a procedure described previously [27]. Briefly, roller tube cultures of monkey kidney cell line MA104 were coinfected at a multiplicity of infection of approximately one with the H-2 strain, FI-14 strain or FI-23 strain and the DS-1 strain. When approximately 75% of the infected cells displayed cytopathic effects, the cultures were frozen and thawed once and the lysate was plated on MA104 cells in a six-well plate (Costar, Corning Inc., Corning, NY) in the presence of G serotype cross-reactive neutralizing monoclonal antibody 57/8 [20] for selection of the desired H-2 × DS-1 and FI-14 × DS-1 and FI-23 × DS-1 reassortants. A plaque displaying a desired gene constellation (i.e., VP4 gene from the H-2, FI-14 or FI-23 strain and the VP7 gene from the DS-1 strain) was plaque purified three times prior to use. Reassortant rotaviruses between equine rotavirus strain H-1 or strain L338 and human rotavirus strain DS-1 were generated in a similar manner except that polyclonal antibodies raised against (i) porcine rotavirus OSU (P[7]G5) strain was used for selection of H-1 × DS-1 (P[7]G2) reassortant and (ii) L338 (P[18]G13) strain was used for selection of L338 × DS-1 (P[18]G2) reassortant. Eagle's minimum essential medium supplemented with 0.5 μg/ml trypsin (Sigma type IX trypsin, Sigma Chemical, St. Louis, MO) and antibiotics was used as maintenance medium and Leibovitz L-15 medium supplemented with antibiotics was employed when making virus dilutions. Agarose (SeaKem ME, BME, Rockland, ME) was used as a solidifying reagent in the overlay medium.

### Rotavirus RNA extraction and PAGE analysis

The standard phenol-chloroform method or TRIzol method was employed to extract rotavirus genomic dsRNA as previously reported [28,29]. Analysis of rotavirus dsRNA was carried out at room temperature (approximately 26°C) in a discontinuous 5%, 7.5%, 10% 12% or 15%, acrylamide resolving slab gel (acrylamide:bisacrylamide 29:1, Bio-Rad Laboratories, Hercules, CA. 18 × 16 × 0.075 cm) with a 3.5% acrylamide stacking gel in the buffer system of Laemmli [30] without SDS using a SE600 gel apparatus (Amersham Biosciences, San Francisco, CA) and Tris-Glycine running buffer (pH 8.3) (Bio-Rad Laboratories). Since the polymerization temperature of acrylamide/bisacrylamide gels has been reported to affect the tertiary structure of the gel thereby influencing electrophoretic mobilities of selected RNA species [31], the polymerization of the PAGE gels used in this study was performed at a single temperature of 37°C in an incubator. In addition, since heat generated during electrophoresis has been reported to affect the mobilities of rotavirus genomic dsRNA [32], a water chiller (Lauda WKL230, Brinkmann Instruments, Westbury, NY) was used, if necessary, to maintain the desired temperature of running buffer especially when evaluating a gel with a high percentage of acrylamide/bisacrylamide. After electrophoresis, viral RNA bands were visualized by staining of the gel with silver nitrate [33].

## Competing interests

The authors declare that they have no competing interests.

## Authors' contributions

All authors read and approved the final manuscript.

LML, XW and ENO carried out the PAGE analyses. YH participated in the design of the study and drafted the manuscript.
